# EphrinA5 protein distribution in the developing mouse brain

**DOI:** 10.1186/1471-2202-11-105

**Published:** 2010-08-25

**Authors:** Claire Deschamps, Milena Morel, Thierry Janet, Guylène Page, Mohamed Jaber, Afsaneh Gaillard, Laetitia Prestoz

**Affiliations:** 1 Institut de Physiologie et Biologie Cellulaires, Université de Poitiers, CNRS, 40 avenue du Recteur Pineau, F-86022, France; 2 GReViC EA3808, Université de Poitiers, CNRS, 40 avenue du Recteur Pineau, F-86022, France

## Abstract

**Background:**

EphrinA5 is one of the best-studied members of the Eph-ephrin family of guidance molecules, known to be involved in brain developmental processes. Using in situ hybridization, ephrinA5 mRNA expression has been detected in the retinotectal, the thalamocortical, and the olfactory systems; however, no study focused on the distribution of the protein. Considering that this membrane-anchored molecule may act far from the neuron soma expressing the transcript, it is of a crucial interest to localize ephrinA5 protein to better understand its function.

**Results:**

Using immunohistochemistry, we found that ephrinA5 protein is highly expressed in the developing mouse brain from E12.5 to E16.5. The olfactory bulb, the cortex, the striatum, the thalamus, and the colliculi showed high intensity of labelling, suggesting its implication in topographic mapping of olfactory, retinocollicular, thalamocortical, corticothalamic and mesostriatal systems. In the olfactory nerve, we found an early ephrinA5 protein expression at E12.5 suggesting its implication in the guidance of primary olfactory neurons into the olfactory bulb. In the thalamus, we detected a dynamic graduated protein expression, suggesting its role in the corticothalamic patterning, whereas ephrinA5 protein expression in the target region of mesencephalic dopaminergic neurones indicated its involvement in the mesostriatal topographic mapping. Following E16.5, the signal faded gradually and was barely detectable at P0, suggesting a main role for ephrinA5 in primary molecular events in topographic map formation.

**Conclusion:**

Our work shows that ephrinA5 protein is expressed in restrictive regions of the developing mouse brain. This expression pattern points out the potential sites of action of this molecule in the olfactory, retinotectal, thalamocortical, corticothalamic and mesostriatal systems, during development. This study is essential to better understand the role of ephrinA5 during developmental topographic mapping of connections and to further characterise the mechanisms involved in pathway restoration following cell transplantation in the damaged brain.

## Background

Ephrins are ligands for transmembrane Eph-receptors, the largest group of receptor tyrosine kinases, that have been shown to be implicated in various developmental mechanisms such as cell adhesion, cell migration, boundary formation, axonal pathӿnding, axon guidance, layer-speciӿc arborisations, target area, topographic mapping and apoptosis [1-5]. A total of 9 members have been identified to date and are divided into two sub-families consisting of 6 ephrinA (A1-A6) and 3 ephrinB (B1-B3) ligand types [5]. EphrinA and B differ in their membrane-anchorage and on their receptor affinity: ephrinA are glycosylphosphatidylinositol (GPI)-linked proteins and bind generally to the EphA-receptors, whereas ephrinB have a transmembrane domain and a cytoplasmic region, and interact preferentially with EphB-receptors. Exceptions in the binding discrimination between classes are that ephrinA5, at high concentration, can bind to EphB2 [6], and ephrinB-ligands to EphA4 [7]. Ephrins and their receptors are highly expressed in the developing nervous system and often in complementary gradients inside delimited regions of the central nervous system [8,9]. This feature is particularly well described in the retinotectal system, where graded Eph and ephrin expressions establish the topographically ordered retinocollicular projection: temporal retinal axons, which express high levels of EphA-receptors, terminate in a low ephrin expression region of the tectum (the anterior part), whereas, nasal axons, which exhibit a low Eph-receptor expression, connect to the posterior tectum, which is a high ephrinA expression region [10].

Within the ephrinA group, ephrinA5 has been extensively studied and was shown to be a ligand for EphA3 [11,12], EphA4 [13,14], EphA5 [10], EphA7 [15] and EphB2 [6] receptors. The study of its expression, mainly explored at the mRNA level in the rodent developing brain, has shown that ephrinA5 is present from early organogenesis [16] to postnatal stages throughout the central nervous system. In the telencephalon, ephrinA5 mRNA is expressed in the olfactory system [17,18], in the lateral and medial ganglionic eminences and their ventricular zones [19-21] and in the cortex [22-27]. EphrinA5 transcript expression has been also detected in the diencephalon (hypothalamus and thalamus) [10,21,27-29] and in the inferior and superior colliculi as well as in the pretectal nuclei and the red nucleus of the mesencephalon [28,30,10,21].

In several systems such as the retinotectal [10,30], the retinothalamic [31] and the thalamocortical [23,24,26,29] ones, ephrinA5 and its receptors have been found to be expressed in opposite gradients on the projections and their target respectively, leading to a repulsive ligand-receptor interaction. An exception to these observations was described in the olfactory system, where high ephrinA5 expressing region is connected by axons containing an important concentration of ephrinA5 receptors. This suggests that ephrinA5 interaction with its receptors could also mediate an attractive signal in some systems [17,32].

Although ephrinA5 mRNA expression has been extensively described during development as mentioned above, distribution of the protein in the developing central nervous system is still lacking. Thereby, putative functions of this molecule during development have been mainly deduced from its mRNA expression pattern and from studies using ephrinA5 knock-out mice. However, the use of these genetic tools may present some limitations, given that, partial redundancy that exists between ephrinA-ligands [33,34].

Given the importance of this guidance membrane-anchored protein location in its actions, sometimes far from the neurone soma expressing the mRNA, we used here immunohistochemistry to analyse the spatiotemporal ephrinA5 protein expression in the mouse brain during embryogenesis and in newborns. We compared the distribution of ephrinA5 protein to the transcript location, and to previous functional studies, providing new insights to the involvement of ephrinA5 in the development of the brain.

## Results

**Figure 1:**
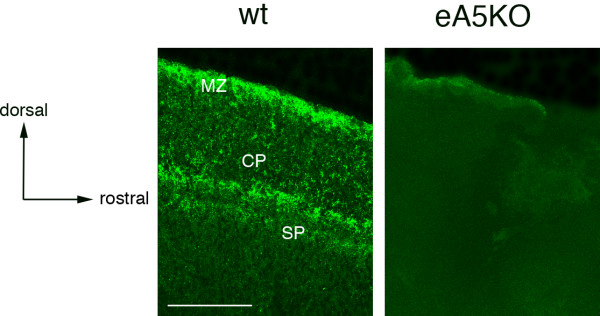
**Anti-ephrinA5 antibody specificity**. EphrinA5 protein expression is represented on sagittal parietal cortex sections of wild type (wt) and ephrinA5 KO (eA5KO) mice. EphrinA5 was strongly expressed particularly in the parietal cortex of wild type mice at E16.5, whereas no staining was observed in the brain of eA5KO mice. *Scale bar: 75 μm. Abbreviations: see list.*

To determine the ephrinA5 protein expression, we used an affinity purified polyclonal antibody directed against a part of the C-terminal region of the human ephrinA5 protein, described in our previous work [21]. Specificity of this antibody was checked comparing immunohistochemistry on sagittal sections of E16.5 wild type and ephrinA5 knock-out (eA5KO) mouse brains. No staining was detected in the eA5KO brains as shown in figure 1 for the parietal cortex where ephrinA5 wild type expression is particularly high.

In wild type mice, we identified the presence of ephrinA5 protein in the mouse brain at four stages during embryonic development: E12.5, E14.5, E16.5, E18.5, and in newborns. Both sagittal and coronal sections showed dynamic spatiotemporal ephrinA5 immunoreactivity in the telencephalic, diencephalic, mesencephalic and metencephalic brain structures. Following P0, the ephrinA5 signal was barely detectable.

Details of ephrinA5 expression distribution are presented below for each developmental stage, and summarized in table 1.

**Table 1:** EphrinA5 protein distribution in the developing mouse brain

	TELENCEPHALON	DIENCEPHALON	MESENCEPHALON	METENCEPHALON
**E12.5**	**• Olfactory nerve** **• Neocortex** **• Piriform cortex** **• LGE** **• LGE dz ***l. v.*➤*m. d.* **• MGE dz ***l. v.*➤*m. d.*	**• Preoptic dz** *l. v.*➤*m. d.* **• Preoptic area** • **Ventral thalamus** *v. r.*➤*d. c.* **• Hypothalamus** *v. r.*➤*d. c.*	**• Mesencephalic tegmentum**	**• **Isthmus **• **Cerebellum

**E14.5**	**• **Olfactory bulb **• Neocortex S1 (CP)** **• Piriform cortex** • **LGE ***l. v. r.*➤*m. d. c.* **• **Subventricular zone	**• Preoptic area** • **Ventral thalamus** *v. r.*➤*d. c.* **• Subthalamic area** •Hypothalamus	**• Mesencephalic tegmentum** • **Superior colliculus** • **Inferior colliculus** *l.*➤*m.* **• Pretectum** *l. v.*➤*m. d.* **• Commissure of superior colliculus**	**• **Isthmus dz **• Cerebellar vermis**

**E16.5**	**• Olfactory structures: GCL, MCL, olfactory tubercle, lateral olfactory tract** • **Septum** • LGE *v. r.*➤*d. c.* **• Neocortex (MZ, CP, SP)** **• Frontal cortex** **• Parietal cortex (S1)** **• Insular cortex** • Retrosplenial cortex **• **Piriform cortex **• **Hippocampus	**• **Preoptic area **• Thalamus** *v. r.*➤*d. c.* **• Hypothalamus**	**• Mesencephalic tegmentum** **• Superior colliculus** • **Inferior colliculus** *c.*➤*r.*	**• **Isthmus **• Cerebellar vermis** • **Pons**

**E18.5**	**• **Olfactory structures: GCL, olfactory tubercle, lateral olfactory tract **• **Striatum **• **Nucleus accumbens **• **Septum *l. v.*➤*m. d.* **• **Lateral migratory stream **• **Piriform cortex **• **Insular cortex **• **Cingulate cortex **• **Retrosplenial cortex **• **Perirhinal cortex **• **Neocortex **• **Parietal cortex (S1) **• **Occipital cortex (layers I & V) **• **Hippocampus (CA1, CA3, DG)	**• **Thalamus *l. d.*➤*m. v.* **• **Hypothalamus	**• **Mesencephalic tegmentum **• **Superior colliculus **• **Inferior colliculus *l.➤m.* **• **Commissure of superior colliculus	**• **Cerebellum **• **Pons

**P0**	**• **Olfactory structures: GCL, subventricular zone, olfactory tubercle, lateral olfactory tract **• **Subventricular zone **• **Striatum *l. v.*➤*m. d.* **• **Nucleus accumbens **• **Septum **• **Parietal cortex **• **Frontal cortex **• **Retrosplenial cortex	**• **Thalamus **• **Hypothalamus	**• **Mesencephalic tegmentum **• **Superior colliculus **• **Inferior colliculus *c.*➤*r.* **• **Commissure of superior colliculus	**• **Cerebellum

EphrinA5 was expressed in the same brain regions of the telencephalon, diencephalon, mesencephalon and metencephalon from E12.5, throughout the embryonic development until P0. The intensity of staining was high from E12.5 to E16.5 and then decreased until P0 to become non-existent in P7 animals (data not shown). The names of brain regions exhibiting a strong ephrinA5 protein expression were underlined and highlighted in bold, whereas names of brain structures showing a moderate ephrinA5 protein expression were only underlined. Names of brain regions exhibiting weak or non-existent ephrinA5 protein expression were not highlighted. Black arrowheads indicate ephrinA5 protein expression gradients. *Abbreviations: see list.*

### EphrinA5 protein expression at E12.5

**Figure 2:**
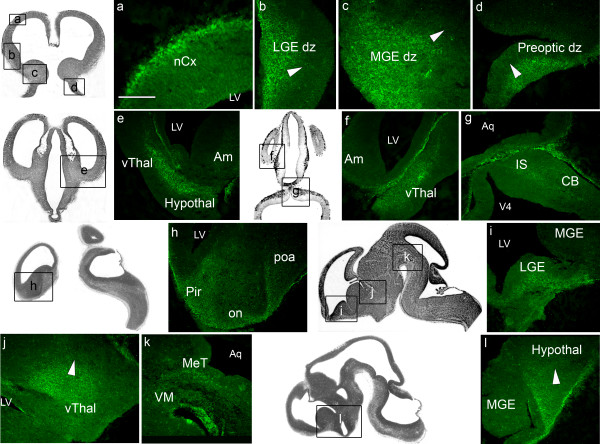
**EphrinA5 protein expression in E12.5 embryonic mouse brain**. EphrinA5 protein distribution is represented on coronal sections in a rostro-caudal sequence (panels a to g), and on sagittal sections in a latero-medial sequence (panels h to l). EphrinA5 protein was strongly expressed in the telencephalon (panels a, b, c, h, i) diencephalon (panels d, e, f, h, j, l) mesencephalon (panel k) and moderately in the metencephalon (panel g). Arrowheads in panel b, c and d indicate a lateral and ventral (high) to medial and dorsal (low) gradient in the lateral and medial ganglionic eminence, and preoptic differentiating zones respectively. In panel j and l, arrowheads show a ventral and rostral (high) to a dorsal and caudal (low) gradient of ephrinA5 protein in the ventral thalamus and hypothalamus respectively. *Scale bar: panels a, c: 75 μm; panel b: 250 μm; panel d: 150 μm; panels e, f, g, h, i, l: 300 μm; panel j: 350 μm; panel k: 200 μm. Abbreviations: see list.*

The earliest stage we examined was embryonic day E12.5 (Figure 2a-l). Within the *telencephalon*, the olfactory nerve (Figure 2h) presented robust ephrinA5 immunoreactivity. Well-stained cell profiles were also observed in the neocortex (Figure 2a) and in the piriform cortex (Figure 2h). Lateral ganglionic eminence (Figure 2i) and lateral (Figure 2b) and medial (Figure 2c) ganglionic eminence differentiating zones exhibited strong ephrinA5 immunoreactivity. In these latter structures, we detected a spatial disparity of ephrinA5 expression in a lateral and ventral (high) to medial and dorsal (low) gradient (arrowheads in figure 2, panels b and c respectively).

Among the *diencephalic structures*, the preoptic differentiating zone (Figure 2d), the preoptic area (Figure 2h), as well as the hypothalamus and the ventral thalamus, visualized on coronal (Figure 2e, f) and sagittal sections (Figure 2j, l), also exhibited strongly labelled ephrinA5 immunoreactive cells. We noticed spatial variations of ephrinA5 expression within the preoptic differentiating zone, the thalamus and the hypothalamus: in the preoptic differentiating zone, ephrinA5 was expressed in a lateral and ventral (high) to medial and dorsal (low) gradient as shown by arrowhead in figure 2, panel d and in a ventral and rostral (high) to dorsal and caudal (low) gradient in the ventral thalamus and in the hypothalamus (arrowheads in figure 2, panels j and l respectively).

In the *mesencephalon*, only the mesencephalic tegmentum exhibited a strong level of ephrinA5 immunoreactivity (Figure 2k). The ventral mesencephalon was unstained (Figure 2k).

The *metencephalon *was moderately stained: ephrinA5 protein was present in the isthmus and in the cerebellum, as visualized in figure 2, panel g.

Overall, at E12.5, ephrinA5 protein was strongly expressed in the telencephalon, diencephalon, mesencephalon, whereas its expression was moderate in the metencephalon (Table 1).

### EphrinA5 protein expression at E14.5

**Figure 3:**
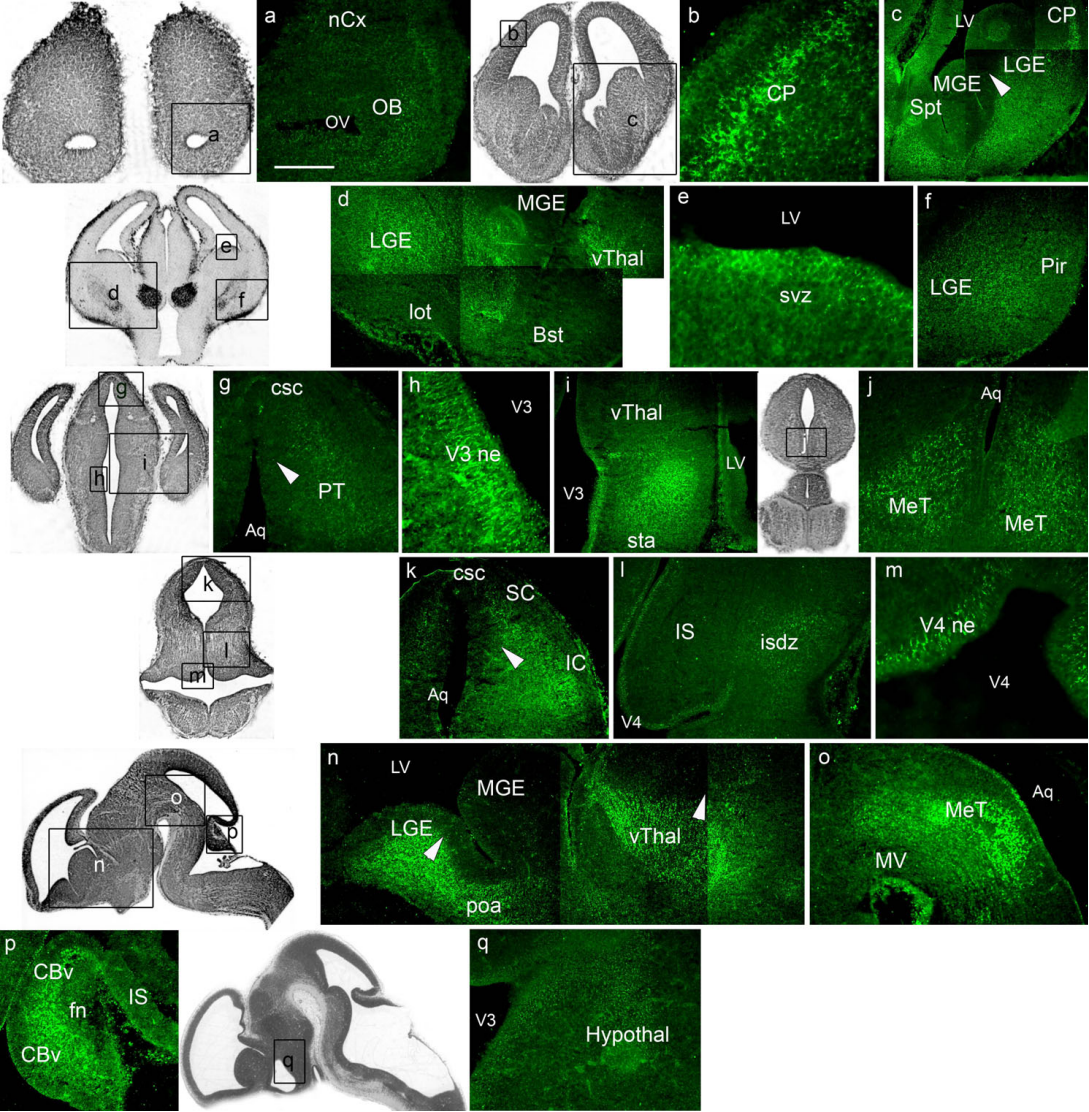
**EphrinA5 protein expression in E14.5 embryonic mouse brain**. EphrinA5 protein distribution is represented on coronal sections in a rostro-caudal sequence (panels a to m) and on sagittal sections in a latero-medial sequence (panels n to q). EphrinA5 protein was differentially expressed within brain regions. EphrinA5 was strongly expressed in the telencephalon (panels b, c, d, f, n) in the diencephalon (panels d, i, n) in the mesencephalon (panels g, j, k, o) and in the metencephalon (panels l and p) excepted in the olfactory bulb (panel a), the subventricular zone (panel e), the hypothalamus (panel q) and the isthmus differentiating zone (panel l) where expression was moderate. Arrowheads in panels c and g indicate ephrinA5 expression gradients respectively in the lateral ganglionic eminence and in the pretectum, in a lateral and ventral (high) to medial and dorsal (low) direction. The inferior colliculus exhibited a lateral (high) to medial (low) gradient (arrowhead in panel k). Arrowheads in panel n show ventral and rostral (high) to dorsal and caudal (low) gradients in the lateral ganglionic eminence and the ventral thalamus. *Scale bar: panels a, b, i, j, k, q: 150 μm; panel c: 300 μm; panel d: 400 μm; panels e, h, m: 75 μm; panels f, g, l, p: 200 μm; panel n: 350 μm; panel o: 250 μm. Abbreviations: see list.*

At E14.5, ephrinA5 expression was strongly expressed in the four major regions of the developing brain (Figure 3a-q). In the *telencephalon*, high level of ephrinA5 immunoreactivity was detected, except in the olfactory bulb (Figure 3a) and in the subventricular zone (Figure 3e) where ephrinA5 protein expression was moderate. Strongly immunoreactive cells were observed in the neocortex, especially in the cortical plate of the primitive somatosensory cortex (Figure 3b) and in the piriform cortex (Figure 3f). EphrinA5 immunoreactivity was also robust in the lateral ganglionic eminence, visualized on coronal (Figure 3c, d, f) and sagittal sections (Figure 3n), in a lateral and ventral (high) to medial and caudal (low) gradient (arrowhead in figure 3, panel c) and in a ventral and rostral (high) to dorsal and caudal (low) gradient (arrowhead in figure 3, panel n).

In the *diencephalon*, ephrinA5 was strongly expressed in the preoptic area (Figure 3n) and in the ventral thalamus, visualized on coronal (Figure 3d) and on sagittal sections in a ventral and rostral (high) to dorsal and caudal (low) gradient (Figure 3n). We also observed a high ephrinA5 staining in the subthalamic area (Figure 3i) and moderate immunoreactivity in the hypothalamus (Figure 3q).

In the *mesencephalon*, coronal (Figure 3j) and sagittal (Figure 3o) sections in the mesencephalic tegmentum exhibited robust ephrinA5 immunoreactivity. Furthermore, ephrinA5 was strongly expressed in the inferior and superior colliculi (Figure 3k) and in the pretectum (Figure 3g). We observed a lateral and ventral (high) to medial and dorsal (low) gradient in the pretectum (arrowhead in figure 3, panel g) and a lateral (high) to medial (low) gradient in the inferior colliculus (arrowhead in figure 3, panel k). The commissure of superior colliculus (Figure 3g, k) also presented robust ephrinA5 immunoreactivity. In return, the ventral mesencephalon was unstained (Figure 3o).

In the *metencephalon*, cells from the isthmus differentiating zone were weakly stained (Figure 3l), whereas the cerebellar vermis exhibited a strong ephrinA5 expression (Figure 3p).

Finally, we noticed that cells from the neuroepithelia of the third (Figure 3h, i) and fourth (Figure 3m) ventricles and of the aqueduct (Figure 3o) exhibited strong ephrinA5 immunoreactivity.

Overall, ephrinA5 protein was strongly expressed in the same brain regions than earlier in the development. The olfactory bulb, the subventricular zone, the hypothalamus and the isthmus differentiating zone were however moderately stained (Table 1).

### EphrinA5 protein expression at E16.5

**Figure 4:**
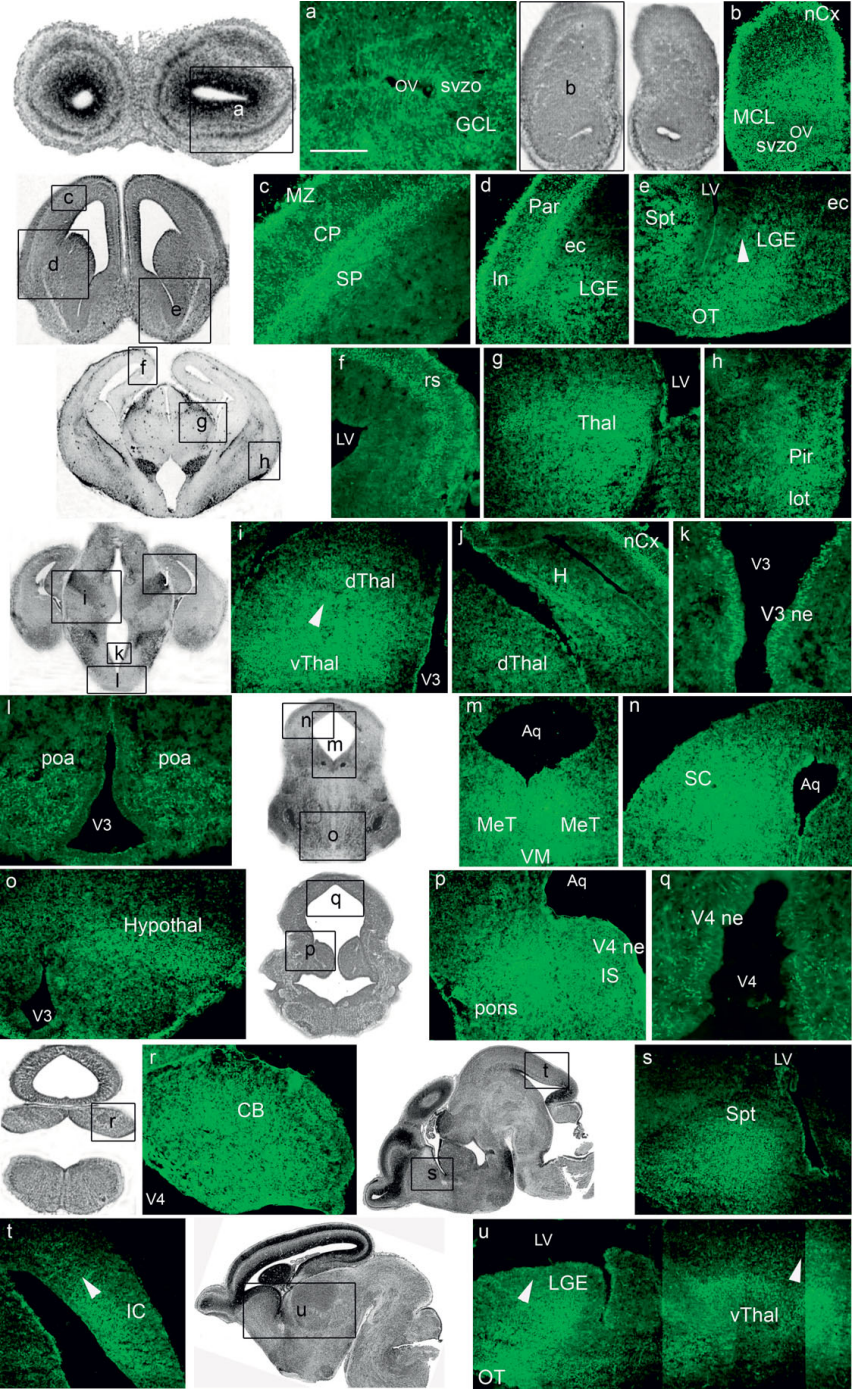
**EphrinA5 protein expression in E16.5 embryonic mouse brain**. EphrinA5 protein distribution is represented on coronal sections in a rostro-caudal sequence (panels a to r) and on sagittal sections in a medio-lateral sequence (panels s to u). At 16.5, ephrinA5 protein was strongly expressed in the telencephalon (panels a, b, c, d, e, j, s, u) diencephalon (panels g, i, j, l, o, u) mesencephalon (panels m, n, t) and metencephalon (panels r, p) excepted in the retrosplenial (panel f) and piriform (panel h) cortices, in the hippocampus (panel j) in the preoptic area (panel l) and in the isthmus (panel p) where the expression was moderate. In the lateral ganglionic eminence, ephrinA5 protein was expressed in a ventral and rostral (high) to dorsal and caudal (low) gradient (arrowheads in panels e and u). In panel i and u, arrowheads represent a ventral and rostral (high) to dorsal and caudal (low) gradient in the thalamus and in panel t, a caudal (high) to rostral (low) gradient in the inferior colliculus. *Scale bar: panels a, k, q: 75 μm; panels b, d, n: 350 μm: panels c, e, o, r: 200 μm; panels f, l, t: 150 μm; panels g, h, i, j, p: 300 μm; panels m, u: 400 μm. Abbreviations: see list.*

At E16.5, the intensity of ephrinA5 immunostaining was high in most of the developing brain structures (Figure 4a-u). The *telencephalon *exhibited strong ephrinA5 expression in olfactory structures such as the granule (Figure 4a) and the mitral (Figure 4b) cell layers of the olfactory bulb, the olfactory tubercle (Figure 4e) and the lateral olfactory tract (Figure 4h). We also detected a high level of ephrinA5 immunoreactivity in the septum (Figure 4s) and in the lateral ganglionic eminence, as visualized on coronal (Figure 4d, e) and sagittal sections (Figure 4u). Within the lateral ganglionic eminence, ephrinA5 was expressed in a ventral and rostral (high) to dorsal and caudal (low) gradient (arrowheads in figure 4, panels e and u). Moreover, the frontal (Figure 4b) and parietal (Figure 4d) parts of the neocortex exhibited a strong ephrinA5 protein expression in the marginal zone, the cortical plate and the subplate as observed in the primitive somatosensory cortex (Figure 4c). Insular (Figure 4d), retrosplenial (Figure 4f) and piriform (Figure 4h) cortices also exhibited ephrinA5 immunoreactive cells, although these two latter structures showed less intense immunostaining. EphrinA5 protein expression was moderate in the hippocampus (Figure 4j).

In the *diencephalon*, ephrinA5 staining in the preoptic area (Figure 4l) was moderate compared to previous stages, whereas the thalamus, visualized on coronal (Figure 4g, i, j) and sagittal sections (Figure 4u), still exhibited strong ephrinA5 expression. In this structure, ephrinA5 staining was higher in the ventral and rostral part than in the dorsal and caudal part (arrowheads in figure 4, panels i and u). An intense staining was also detected in the hypothalamus (Figure 4o).

In the *mesencephalon*, the mesencephalic tegmentum (Figure 4m) and the superior colliculus (Figure 4n) exhibited strongly immunostained cells. EphrinA5 labeling was also detected in the ventricular zone of the inferior colliculus in a caudal (high) to rostral (low) gradient (arrowhead in figure 4, panel t). The ventral mesencephalon was unstained (Figure 4m).

In the *metencephalon*, the isthmus was moderately stained (Figure 4p) whereas high immunoreactivity in the cerebellum (Figure 4r) and in the pons (Figure 4p) was detected.

Neuroepithelia of the third (Figure 4k) and fourth (Figure 4p, q) ventricles and neuroepithelium of the aqueduct (Figure 4m, n) exhibited ephrinA5 immunostaining as well.

**Figure 5:**
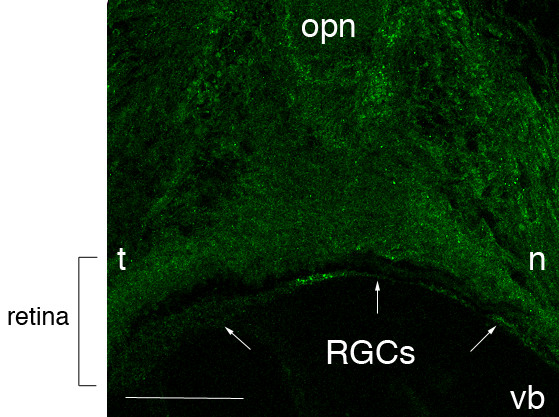
**EphrinA5 protein expression in E16.5 embryonic mouse retina**. EphrinA5 protein distribution is represented on horizontal section of mouse retina. EphrinA5 is expressed in the nasal part of the retina and retinal ganglion cells. *Scale bar: 130 μm. Abbreviations: see list.*

Moreover, to specify retinocollicular mapping, we tested the expression of ephrinA5 protein in the retina. Immunohistochemistry localized ephrinA5 protein in the nasal part of the retina and the retinal ganglion cell layer (Figure 5).

Overall, E16.5 embryonic stage showed high levels of ephrinA5 immunoreactivity in the same regions than those previously described earlier in the development, with the exception of the preoptic area that was moderately stained compared to E12.5 and E14.5 stages (Table 1).

### EphrinA5 protein expression at E18.5

**Figure 6:**
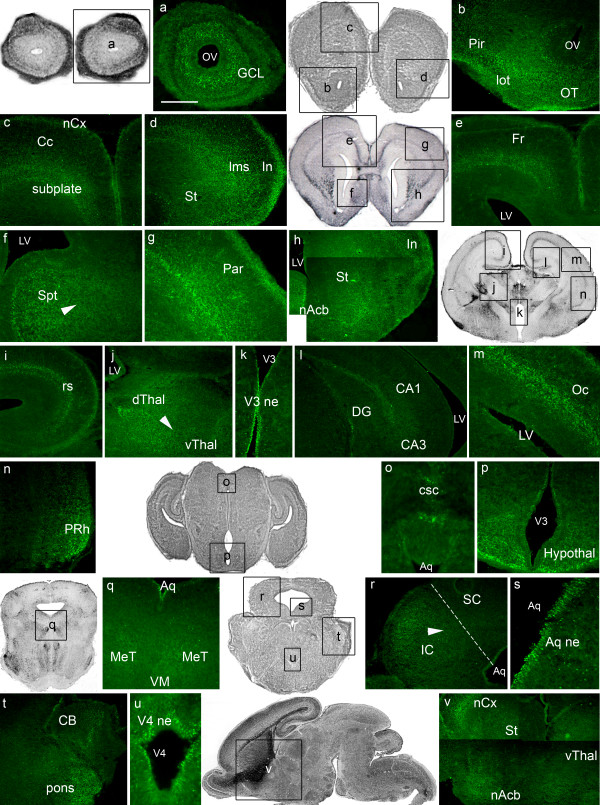
**EphrinA5 protein expression in E18.5 embryonic mouse brain**. EphrinA5 protein distribution is represented on coronal sections in a rostro-caudal sequence (panels a to u) and on a sagittal section (panel v). At E18.5, ephrinA5 is mostly expressed in the telencephalon especially in olfactory structures (panels a, b) in the septum (panel f) in the lateral migratory stream (panel d) and in the cortex (panels g, m, n, v) at a moderate level. The thalamus (panel j) and inferior colliculus (panel r) showed also a moderate expression, whereas other brain structures exhibited weak ephrinA5 immunoreactivity. Arrowheads in panels f, j and r represent respectively a lateral and ventral (high) to medial and dorsal (low) gradient in the septum, a lateral and dorsal (high) to medial and ventral (low) gradient in the thalamus and a lateral (high) to medial (low) gradient in the inferior colliculus. Dotted line in panel r indicates the limit between the superior and the inferior colliculi. *Scale bar: panels a, b: 250 μm; panels c, e, n: 400 μm; panels d, j, r, t: 300 μm; panels f, g, l: 200 μm; panel h: 450 μm; panels i, m: 250 μm; panel k: 100 μm; panel o: 110 μm; panel p: 175 μm; panel q: 150 μm; panel s: 100 μm, panel v: 550 μm. Abbreviations: see list.*

At E18.5, ephrinA5 protein expression was generally less intense than earlier in the development (Figure 6a-v). In the *telencephalon*, the granule cell layer of the olfactory bulb (Figure 6a), the olfactory tubercle and the lateral olfactory tract (Figure 6b) exhibited a moderate immunoreactive intensity. Moreover, ephrinA5 expression was weak in the striatum as shown on coronal (Figure 6d, h) and sagittal sections (Figure 6v), and almost non-existent in the nucleus accumbens (Figure 6v). A moderate ephrinA5 expression was detected in the septum in a lateral and ventral (high) to medial and dorsal (low) gradient (arrowhead in figure 6, panel f) and in the lateral migratory stream (Figure 6d). The piriform (Figure 6b), insular (Figure 6d, h), cingulate (Figure 6c), including the retrosplenial cortex (Figure 6i), and the frontal cortex (Figure 6e) exhibited weak staining intensity. Moderate intensity of ephrinA5 expression was observed in the perirhinal cortex (Figure 6n), in layers I and V in the occipital cortex (Figure 6m) and in the primary somatosensory region of the parietal cortex (Figure 6g). In the hippocampus, weak immunostaining was localized in the CA1 and CA3 regions, and in the dentate gyrus (Figure 6l).

In *diencephalic *structures, ephrinA5 expression was moderate in the thalamus, with the strongest immunoreactivity in the dorsal part, contrary to the previous stages, thus leading to a lateral and dorsal (high) to medial and ventral (low) gradient (arrowhead in figure 6, panel j). Weakly stained cells were detected in the hypothalamus (Figure 6p).

In the *mesencephalon*, the commissure of the superior colliculus (Figure 6o), the mesencephalic tegmentum (Figure 6q) and the superior and inferior colliculi (Figure 6r) exhibited ephrinA5 immunoreactive cells. EphrinA5 expression was however stronger in the inferior part of the colliculus than in the superior region, exhibiting a lateral (high) to medial (low) gradient (arrowhead in figure 6, panel r). The ventral mesencephalon was unstained (Figure 6q).

In the *metencephalon*, the cerebellum and the pons were weakly stained (Figure 6t).

Finally, cells from the neuroepithelia of the third (Figure 6k, p) and fourth (Figure 6u) ventricles, and the neuroepithelium of the aqueduct (Figure 6q, r, s) exhibited high immunoreactivity.

### EphrinA5 protein expression at P0

**Figure 7:**
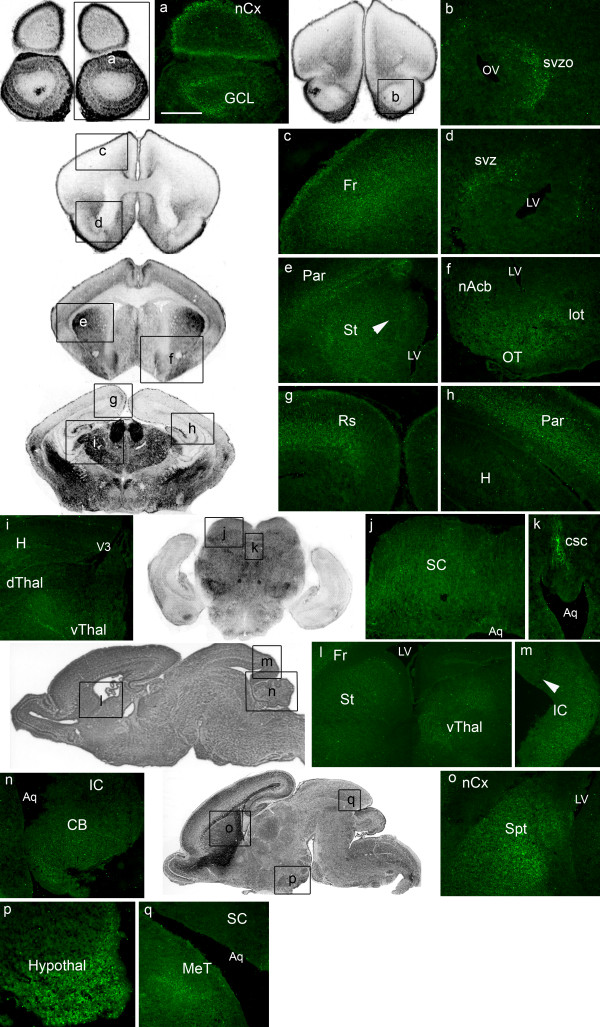
**EphrinA5 protein expression in newborn mouse brain**. EphrinA5 protein distribution is represented on coronal sections in a rostro-caudal sequence (panels a to k) and on sagittal sections in a medio-lateral sequence (panels l to q). EphrinA5 protein expression dramatically decreased in all brain regions compared to previous stages. The strongest expression was observed in the septum (panel o) the hypothalamus (panel p) and in the commissure of superior colliculus (panel k). Arrowheads in panels e and m indicate respectively a discrete lateral and ventral (high) to medial and dorsal (low) gradient in the striatum and a caudal (high) to rostral (low) gradient in the inferior colliculus. *Scale bar: panel a: 250 μm; panels b, m, p, q: 200 μm; panels c, e, f, g, h, n: 300 μm; panels d, k: 150 μm; panels i, l: 500 μm; panel j: 400 μm; panel o: 350 μm. Abbreviations: see list.*

In newborn mice, ephrinA5 expression intensity was dramatically decreased in all regions of the brain, compared to the previous stage E18.5 (Figure 7a-q). In the *telencephalon*, we were however able to detect weakly stained cells in the olfactory structures: the granule cell layer (Figure 7a), the olfactory subventricular zone (Figure 7b), the olfactory tubercle (Figure 7f), and the lateral olfactory tract (Figure 7f). Moreover, ephrinA5 immunoreactive cells were observed in the subventricular zone (Figure 7d), in the striatum (Figure 7e, l), and in the nucleus accumbens (Figure 7f). In the striatum, we detected a discrete ephrinA5 expression gradient from the lateral and ventral (high) to the medial and dorsal (low) parts (arrowhead in figure 7, panel e). Moderate ephrinA5 staining was observed in the septum (Figure 7o) whereas the parietal (Figure 7e, h), the frontal (Figure 7c) and the retrosplenial (Figure 7g) cortices exhibited weak ephrinA5 protein expression. The hippocampus (Figure 7h), the piriform, the occipital and the perirhinal cortices were unstained (data not shown).

Within the *diencephalic *structures, a very weak ephrinA5 signal was present in the dorsal and in the ventral part of the thalamus (Figure 7i, l). The hypothalamus expressed moderate ephrinA5 protein expression level (Figure 7p).

In the *mesencephalon*, the mesencephalic tegmentum (Figure 7q) exhibited weakly immunostained cells as well as the superior (Figure 7j) and inferior (Figure 7m) colliculi. In the inferior colliculus, we however detected a caudal (high) to rostral (low) gradient (arrowhead in figure 7, panel m). The commissure of superior colliculus exhibited moderate ephrinA5 immunoreactivity (Figure 7k).

Within the *metencephalon*, weak ephrinA5 immunoreactivity was detected in the cerebellum (Figure 7n).

Ventricles and Sylvius aqueduct's neuroepithelia were unstained (data not shown).

Overall, at P0, ephrinA5 protein expression decreased in all brain regions compared to previous stages. The strongest ephrinA5 protein expression was found in the septum, in the hypothalamus and in the commissure of superior colliculus, although these protein expression levels were still moderate compared to the earliest developmental stages (Table 1).

Following P0, ephrinA5 expression was barely detectable.

## Discussion

The present work describes for the first time, the distribution of ephrinA5 protein in the embryonic developing mouse brain. EphrinA5 protein expression appeared to be dynamic and complex, and particularly high from E12.5 to E16.5 in the olfactory bulb, the cortex, the LGE/striatum, the thalamus, and the colliculus. Its expression generally decreased from E18.5 to become very weak at P0 and extinct at P7 (data not shown), contrary to the mRNA level that stays elevated in the newborn [9]. In spite of this graded decrease, ephrinA5 protein expression at P0 was still observed in the same regions of the brain than at earlier stages (i.e. in the olfactory structures, cortex, striatum, thalamus, hypothalamus, nucleus accumbens, colliculus and cerebellum). As primary molecular events in topographic map formation occur between E15 and P7 in the mouse [49], this suggests that high ephrinA5 expression during embryogenesis, may participate in early target connection mechanisms, whereas ephrinA5 may be implicated to a lesser extent in map remodelling process during the first postnatal week. This transient expression during embryonic development is in line with early axon guidance and topographic mapping in the olfactory, the retinotectal, the thalamocortical, the corticothalamic and the mesostriatal systems. This indicates multiple functions of this molecule in the establishment of these systems, as discussed in the following sections.

### Central olfactory system

To the best of our knowledge, the olfactory system is the only brain system, in which ephrinA5 expression has been studied at the protein level, using immunohistochemistry [35]. Our study is complementary to this one as it brings new data especially concerning the expression of the protein in the secondary olfactory neuron population.

Olfaction initiates in the nasal neuroepithelium where primary olfactory neurons reside, each one encoding a single odour receptor type [36,37]. These neurons send axons, forming the olfactory nerve and project to the olfactory bulb in the rostro-ventral telencephalon, where they sort out and target specific glomeruli. Glomeruli are designed as contact zone between growth cones of the primary olfactory neurons and dendrites of secondary olfactory neurons, known as mitral and tufted cells located in the olfactory bulb [35]. Primary olfactory neurons, mitral and tufted cells are neurons supporting early synaptic relays of the mouse olfactory system and so arise in a relatively parallel fashion between E9.5 and E10.5 [38]. In our study, we showed for the first time that the olfactory nerve expressed ephrinA5 in early embryogenesis (E12.5), suggesting either an involvement of this molecule in the guidance of primary olfactory neurons into the olfactory bulb or, an implication of ephrinA5 in the fasciculation of primary olfactory axons inside the olfactory nerve, possibly through an interaction with EphA4- and/or EphA5-receptors, shown to be expressed during embryogenesis in the olfactory nerve [35].

Interestingly, we found that ephrinA5 was expressed in the mitral cell layer of the olfactory bulb, transiently during embryogenesis, at E16.5. The function of this molecule at this precise stage is still unclear but it may be implicated in the positioning of the mitral cell layer in the olfactory bulb. Indeed, ephrins are known to be involved in cell migration and boundary formation in other nervous systems [2,3], and at this stage the cell body of developing mitral cells appears to transform from tangential to radial orientation with rearrangement of its microtubules structure [39]. Moreover, the presence of potential ephrinA5 receptors such as EphA3 [40], EphB2 [41], EphA5 [35,42,43], EphA7 and EphA4 [35] has been also described in the mitral cells during embryogenesis. This suggests that ephrinA5 and its receptors are not expressed in complementary gradient as it has been described for other systems such as the retinotectal one [8,44]. Instead, we showed that ephrinA5 is uniformly expressed in this structure as for its receptors [40-42,35,20]. This ligand-receptor interaction may result in an attraction of the cells, thus regulating the connections of mitral cells. Indeed, this mechanism has already been demonstrated in the accessory olfactory system, where axons expressing high levels of ephrinA5 project to regions of the accessory olfactory bulb that express high levels of EphA6, suggesting that EphA6-ephrinA5 interaction promotes adhesion or attraction between first and second order accessory neurones [17].

Later during embryogenesis, the mitral cells leave the olfactory bulb in the lateral olfactory tract, which synapses on five major regions of the mature telencephalon including the olfactory tubercle and the piriform cortex that project to the thalamus. We detected the expression of ephrinA5 protein from E16.5 on in the lateral olfactory tract and the olfactory tubercle. This expression was strong at E16.5, slightly diminished at E18.5 and became very weak at P0, suggesting that this molecule may be involved in the secondary olfactory neuron guidance, since its presence is concomitant to the development of this telencephalic pathway [45]. EphrinA5 expression in the lateral olfactory tract and olfactory tubercle may also have a role in the fasciculation of axons inside these structures, since fasciculation can be regulated by manipulating chemorepulsive interactions mediated by Eph receptors [46]. As axon pathfinding in olfactory map formation has been mainly investigated when primary olfactory axons enter the olfactory bulb glomeruli [47], our data give new insights concerning the guidance of the secondary mitral cell axons onto the thalamus.

Finally, we detected the presence of ephrinA5 protein in the piriform cortex at E12.5, earlier than the expression of this protein in the mitral cells and in the lateral olfactory tract that appeared at E16.5. This ephrinA5 expression in the piriform cortex dramatically decreased from E18.5, when the secondary olfactory axons connect this target [45]. This suggests that ephrinA5 protein expression in the piriform cortex during early embryogenesis may not be related to the connection of the axons of the lateral olfactory tract, but rather may play a role in the formation of the piriform cortex architecture.

### Visual system

Expression of ephrinA5 transcript has been extensively studied in this system and especially in the retinocollicular topographic mapping [10,48-50]. We investigated ephrinA5 protein expression in the retina and detected a high expression in the nasal part compared to the temporal one. This result is in accordance with the previously described retinal ephrinA5 expression pattern in the chick [51] and with ephrinA5 mRNA expression found in a high-nasal-to-low-temporal gradient in the developing mouse embryo retina [52]. As previously described, the mouse retinal ganglion cells also expressed EphA5 and EphA6 receptors [53]. This suggests that ephrinA5 protein may be co-expressed with these molecules and interact in *cis *or in *trans *then modulating the tectum or colliculus connectivity. Indeed, several studies confirmed the role of axonally expressed ephrinA ligands in the development of the retinotectal projection [54]. However, whether growth cones switch between *cis *and *trans *configuration or *cis *and *trans *configurations occur in parallel in the same growth cone, is still unclear.

As our findings in the retina, the results of our analysis in the central visual system were generally consistent with the findings of the past surveys describing the mRNA distribution of ephrinA5 in the 3 main targets of retinal ganglion cell axons: the thalamus, the pretectum, and the superior colliculus. Indeed, we showed that ephrinA5 protein is strongly expressed in these structures especially from E14.5 to E16.5 (Table 1), when the axons of the optic tracts reach their final destinations in the superior colliculus at E14 [30], and in the thalamic dorsal lateral geniculate nuclei at E16 [10]. This is in accordance with an implication of ephrinA5 in the topographic mapping of the visual connections especially in the superior colliculus [48-50]. It has to be noticed however, that ephrinA5 mRNA expression was described as soon as E12.5 in the developing superior colliculus [28,30], whereas we did not detect ephrinA5 protein in this structure earlier than E14.5. This suggests that ephrinA5 may be implicated in this topographic mapping only from E14.5, when the retinal connections reach the superior colliculus. On the contrary, in the thalamus and in the mesencephalic tegmentum, ephrinA5 protein level was elevated from E12.5 onwards. This expression seems to be too precocious to involve ephrinA5 in visual connection mapping and more probably may be implicated in the formation of other systems involving the thalamus, such as the thalamocortical system as discussed below, and the mesencephalic tegmentum, such as the rubrospinal system.

The general high ephrinA5 expression during embryogenesis, may highlight a role for ephrinA5 in the initial diffuse projection when retinal ganglion cells substantially overshoot their appropriate target by E16 along the antero-posterior axis of the superior colliculus [48] and at a lesser extent later on, when map remodelling occur during the first postnatal week.

EphrinA5 RNA is known to be expressed in a caudal (high) to rostral (low) gradient in the mouse superior colliculus [44,50,55]. This gradient is thought to be at the basis of the chemorepellent action of ephrinA5 on retinal ganglion cell's axons arising from the nasal part of the retina [50]. However, at the protein level, ephrinA5 expression was homogenous in the superior colliculus without any detectable expression gradient. As suggested by Greferath *et al*. [56] concerning EphA4 receptor expression in the striatum, this may be due to the fact that the antibody localizes ephrinA5 protein in both cell bodies and axons bundles, whereas RNAs are concentrated in the cell somas. Moreover, studies describing the EphA5 protein expression in the developing mouse [43] did not detect any protein gradient expression.

Among studies exploring the expression of EphA-receptors at the protein level in the developing mouse brain, some showed that the potential ephrinA5 receptors EphA4 and EphA5, are expressed in the central visual system: EphA4 protein is present in the thalamus and superior colliculus from E11 to P6; and in the pretectal nucleus from E15 to P6 [56]. EphA5 protein is expressed in the geniculate nucleus of the thalamus from E17 as well as in the pretectum and the superior colliculus from E9 to E17 [43]. Together with our present work, these findings suggest a concomitant expression of ephrinA5 with (1) EphA4 and EphA5 in the superior colliculus, (2) EphA4 in the thalamus and (3) EphA5 in the pretectum. Moreover, a detailed analysis of protein gradients inside these regions may help to understand their interactions and their chemorepulsive or chemoattractive actions. However, it is unlikely that the co-expression of ligand and receptor in the same region of the visual system is responsible of an attractive mechanism, since it has been extensively shown, using in vitro studies and double ephrinA2/ephrinA5 knock-out mice [30,48-50], that the topographic map of the visual system is established by a chemorepellent activity of ephrinAs [50]. Nevertheless, it may be that ligands and receptors are present on the same cells, as it has been shown in the chick retinal ganglion cells, where ephrinA2, ephrinA5 and EphA5 mRNA are co-expressed [51]. In this case, the presence of ephrinA-ligand on the retinal ganglion cells could control the EphA-receptor function through a modulation of its intracellular signalling pathway [51] as discussed above.

### Cortex and thalamocortical projections

We detected ephrinA5 protein in the developing cortex from E12.5 to E18.5 with an expression pattern becoming more complex as cortical layers were forming. We found a particularly strong signal at E16.5 in the primitive primary somatosensory cortex (S1), when thalamic axons reach the subplate zone. This is consistent with the presence of ephrinA5 mRNA in the subplate and cortical plate of S1 at this stage [22-24,27]. Indeed, ephrinA5 is thought to interact with and repulse ventrobasal thalamic terminal arbors expressing EphA4, and to be responsible for the precise topographic mapping of thalamic afferents into S1 [24]. Moreover, we showed a strong ephrinA5 protein expression in other regions of the developing cortex, especially in the parietal, frontal and insular cortices at E16.5, suggesting that ephrinA5 may be involved in the refinement of other thalamocortical projections and probably through an interaction with 3 potential receptors: EphA3, EphA4 and/or EphA5 that have been detected in these regions during mouse embryogenesis. Indeed, EphA3 protein is expressed in the cortical plate and cortical intermediate zone from E12 to P0, especially in thalamocortical axons [40]. EphA4 protein is expressed from E11 to P6 in the developing cortex [56], and EphA5 protein is present in the intermediate zone and cortical plate [43] whereas its transcript is expressed in the dorsal and medial thalamus that connect to the developing cortex [57], suggesting an expression of EphA5 protein in the thalamocortical projections.

### Thalamus and corticothalamic projections

Previous studies showed different patterns of ephrinA5 mRNA expression, depending of the thalamic nuclei and the stage observed: at E13.5, Bolz *et al. *[27] described a weak mRNA expression in the ventricular zone of the dorsal thalamus at the caudal level with no detectable expression in the mid- or rostral levels of the dorsal thalamus. Feldheim *et al. *[10] described ephrinA5 mRNA expression from E14.5, in the dorsal and ventral lateral geniculate nuclei in a ventral, lateral and anterior (high) to dorsal, medial and posterior (low) gradient. Between E16.5 and E18.5, nuclei of the lateral part of the dorsal thalamus [27] and ventral nuclei [29] both exhibit ephrinA5 mRNA. Our results partially match with these previous studies, as we showed here that ephrinA5 protein was strongly expressed in the thalamus in a ventral and rostral (high) to dorsal and caudal (low) gradient especially from E12.5 to E16.5. Interestingly, we observed that this gradient inverted from E16.5, when cortical axons connect the thalamus [58]. This is consistent with an implication of ephrinA5 in the corticothalamic projection establishment as proposed by Torii and Levitt [59] who hypothesized that the corticothalamic projections are directed by ephrinA-EphA signalling and more precisely that axons from sensory cortex find a precise target in the thalamus by responding to local levels of ephrinA5. In this region, ephrinA5 may interact with EphA4 protein, described in the thalamus from E13.5 to postnatal stages [56] to ensure the formation of terminal corticothalamic arbors.

### Mesostriatal system

EphrinA5 protein was strongly expressed in the main target structure of the ventral mesencephalic axons: the lateral ganglionic eminence and later in development, the striatum. As described for mRNA levels [19,21], this expression was high from E12.5 to E16.5 and then diminished later in development. We observed a graduated ephrinA5 protein expression in a ventral and rostral (high) to a dorsal and caudal (low) gradient that was not spatially concordant with the ephrinA5 RNA expression, described as not graded in developing striatum [60,19,21]. As we discussed in a previous work, it is likely that the presence of this ephrinA5 protein expression gradient helps mesencephalic neuron subpopulations to differentially connect the dorsal and ventral parts of the striatum during development [21].

No ephrinA5 protein was detected in the ventral mesencephalon from E12.5 to P0, suggesting that this protein was present exclusively at the target site of the mesotriatal projections, when the mesencephalic axons reach the LGE [61]. This strongly suggests that ephrinA5 may be involved in the refinement of mesencephalic arbors in the striatum especially by interacting with EphA5, expressed in the LGE between E13 and E17 [43] and in the ventral mesencephalon in the dopaminergic mesencephalic cells projecting to the LGE [21]. Finally, the implication of ephrinA5 in the mesencephalic projections onto the striatum is in accordance with previous work showing that disruption of EphA/ephrinA interactions resulted in the mistargeting of a fraction of mesencephalic dopaminergic projections [62].

## Conclusions

In the present study we provide the first detailed description of ephrinA5 protein distribution in the embryonic developing mouse brain. We found that ephrinA5 is strongly expressed from E12.5 to E16.5, in the olfactory bulb, the cortex, the striatum, the thalamus, and the colliculi, suggesting its implication in topographic mapping of olfactory, retinotectal, thalamocortical, corticothalamic and mesostriatal systems. These new data may help to characterise the molecular basis of pathfinding of these systems, during development. Moreover, given the importance of developmental processes in brain repair, these results may open new tracks in the improvement of pathway restoration following cell transplantation in the damaged brain.

## Methods

### Animals

Housing of the animals and all animal experimental procedures were carried out in accordance with the guidelines of the French Agriculture and Forestry Ministry (decree 87849) and the European Communities Council Directive (86/609/EEC). All efforts were made to reduce the number of animals used and their suffering.

Embryonic and postnatal C57Bl/6 wild type mice of different developmental stages (E12.5, E14.5, E16.5, E18.5, P0) were used in this study. E0.5 was defined as the plug date, and P0 as the date of birth. Moreover, E16.5 embryonic eA5KO mice were used to test the anti-ephrinA5 antibody specificity. These mice were kindly provided by Dr. P. Vanderhaeghen (Belgium).

### Genotyping of eA5KO mice

Mice genomic DNA was extracted from 5 mm-long tail fragments. The tissue was digested in an alkaline lysis solution (25 mM NaOH, 0.2 mM Na_2_-EDTA, pH = 12) at 94°C for 1 h. The lysate containing the genomic DNA was then neutralized by the addition of 40 mM Tris-HCl, pH5.

Amplification was performed using the PCR AccuPrime Taq DNA Polymerase System (Invitrogen) according to the protocol provided by the manufacturer. Sense primer for ephrin-A5 is 5'-TCC AGC TGT GCA GTT CTC CAA AAC A-3 '. Antisense primers for the detection of ephrin-A5 wild type and mutant alleles are respectively 5 '-ATT CCA AGA GGG GTG ACT ACC ACA TT-3' and 5'-AGC CCA GAA AGC GAA GGA GCA AAG C-3 '. They were designed to generate PCR fragments of 397 and 513 bp respectively. After an initial step of DNA denaturation at 94°C for 5 min, amplification was carried out for 30 cycles at 94°C for 1 min, 46.5°C for 1 min and 72°C for 1 min. A final step of 5 min at 72°C was performed.

After separation by electrophoresis on 2% agarose gels, the size of the PCR products obtained allowed us to distinguish between the wild-type ephrin-A5(+/+) animals (one PCR product at 397 bp) from mutated animals, homozygous ephrin-A5 (-/-) (one PCR product at 513 bp) and heterozygous ephrin-A5 (+/-) (two PCR products at 397 and 513 bp).

### Antibodies

Primary antibody directed against the C-terminal domain of ephrinA5 has been reported before [21]. This is a polyclonal goat anti-ephrinA5 antibody (sc-6075, Santa Cruz Biotechnology, Santa Cruz, USA) that recognizes a 19 amino acid epitope that falls in the last 50 amino acids of the human ephrinA5 protein. This antibody was used at a dilution of 1:100. Secondary biotinylated rabbit anti-goat antibody was purchased from Vector (Burlingame, CA) and used at a dilution of 1:200.

Specificity of this antibody was checked comparing immunohistochemistry in wild type and homozygous ephrinA5KO mice.

### Immunohistochemistry

Pregnant wild type and eA5KO mice were cervically dislocated at different developmental stages. Embryonic (E12.5, E14.5, E16.5, E18.5) and newborn (P0) wild type brains were dissected out and E16.5 wild type embryo heads containing the eyes were taken. Homozygous E16.5 eA5KO embryo brains were taken to check the specificity of the anti-ephrinA5 antibody. These tissues were fixed in 4% PFA overnight and cryoprotected with 30% sucrose in 0.1 M phosphate buffered saline (PBS) for 48 h at 4°C, excepted for the E16.5 embryo heads that were dehydrated in alcool and butanol after fixation. Two brains of each developmental stage were then frozen in cold isopentane and cut on a cryostat into sagittal and coronal 20 μm-thick sections. Sections were stored at -80°C until immunohistochemistry processing. Dehydratation of heads, consisting of successive baths of twice 1 hr each in 70°, 95°, 100°alcool, butanol and butanol/paraffin (v/v), were embedded in paraffin and cut on a microtome into horizontal 10 μm-thick sections. Before immunohistochemistry, paraffin sections were deparaffinized in toluene solution three time for 5 min and rehydrated twice in successive alcool baths of 100°, 90°, 70°, 30°and distilled water for 5 min each.

Immunohistochemistry was performed as previously described in Deschamps *et al*, [21]. Briefly, the sections were rehydrated in 0.1 M potassium phosphate buffered saline (KPBS) (0.9% NaCl, 0.08 M K2HPO4, 0.02 M KH2PO4), and then post-fixed with 4% PFA for 2 h at room temperature (RT). After several washes with 0.1 M KPBS, sections were incubated with a blocking solution (5% rabbit serum, 0.25% Triton-X100 in 0.1 M KPBS) for 3 h at RT to saturate non-specific sites. The anti-ephrinA5 antibody was then applied on sections overnight at 4°C. After several washes with 0.1 M KPBS, the sections were incubated with the secondary biotinylated antibody for 1 h 30 at RT. After extensive washing with 0.1 M KPBS, ephrinA5 expression was visualized with neutravidin™ fluorescein conjugate (Molecular Probes, Eugene, USA), diluted at 1/200 in blocking solution, for 45 min at RT. Following several washes with 0.1 M KPBS, slides were covered with mowiol anti-fade mounting medium [63,64].

### Immunostaining analysis

Sections were observed and photographed with a microscope (Olympus BX60F5) and camera setting (SPOT32, Diagnostic instruments, inc). Image brightness and contrast were adjusted with Adobe Photoshop in the same conditions for all pictures. Brain structures were identified using brain mouse atlases [65,66].

## Abbreviations

Am: amygdala; Aq: Sylvius acqueduct; Aq ne: Sylvius acqueduct neuroepithelium; Bst: bed nucleus of stria terminalis; c: caudal; CA1, 3: hippocampal area CA1, 3 (Ammon's Horn); CB: cerebellum; CBv: cerebellar vermis; Cc: cingulate cortex; CP: cortical plate; d: dorsal; DG: dentate gyrus; dThal: dorsal thalamus; ec: external capsule; eA5KO: ephrinA5 knock-out; fn: fastigial nucleus; Fr: frontal cortex; GCL: granule cell layer; H: hippocampus; Hypothal: hypothalamus; IC: inferior colliculus; In: insular cortex; IS: isthmus; isdz: isthmus differentiating zone; KPBS: potassium phosphate buffered saline l: lateral; LGE: lateral ganglionic eminence; LGE dz: lateral ganglionic eminence differentiating zone; lms: lateral migratory stream; lot: lateral olfactory tract; LV: lateral ventricle; m: medial; MCL: mitral cell layer; MeT: mesencephalic tegmentum; MGE: medial ganglionic eminence; MGE dz: medial ganglionic differentiating zone; MZ: marginal zone; n: nasal; nAcb: nucleus accumbens; nCx: neocortex; OB: olfactory bulb; Oc: occipital cortex; on: olfactory nerve; opn: optic nerve; OT: olfactory tubercle; OV: olfactory ventricle; Par: parietal cortex; PB: phosphate buffer; PBS: phosphate buffered saline; PFA: paraformaldehyde; Pir: piriform cortex; poa: preoptic area; Preoptic dz: preoptic differentiating zone; PRh: perirhinal cortex; PT: pretectum; r: rostral; RGCs: retinal ganglion cells; Rs: retrosplenial cortex; RT: room temperature; SC: superior colliculus; csc: commissure of superior colliculus; SP: subplate; Spt: septum; St: striatum sta: subthalamic area; svz: subventricular zone; svzo: olfactory subventricular zone; S1: primary somatosensory cortex; t: temporal; Thal: thalamus; v: ventral; vb: vitreous body VM: ventral mesencephalon; vThal: ventral thalamus; V3: third ventricle; V3 ne: neuroepithelium of the third ventricle; V4: fourth ventricle; V4 ne: neuroepithelium of the fourth ventricle; wt: wild type

## Authors' contributions

CD carried out the immunoassays and the image captures, helped in western-blot assays and genotyping procedures, participated to the analyses and interpretations of data, and wrote the manuscript. MM and GP carried out western-blot assays. TJ carried out genotyping procedures. MJ and AG contributed to the conception and design of the data, and revised critically the manuscript. LP designed and coordinated the study, wrote the manuscript, and participated to the analyses and interpretations of data. All authors read and approved the final manuscript.
